# Active tuberculosis in household contacts of bacteriologically confirmed pulmonary tuberculosis patients: A multicenter study finding the ‘Missed One’ in Central Ethiopia

**DOI:** 10.1371/journal.pone.0316903

**Published:** 2025-02-18

**Authors:** Getachew Seid, Ayinalem Alemu, Getu Diriba, Michael Hailu, Amanuel Wondimu, Mengistu Tadesse, Gemechu Tadesse, Solomon H Mariam, Balako Gumi

**Affiliations:** 1 Ethiopian Public Health Institute, Addis Ababa, Ethiopia; 2 Aklilu Lemma Institute of Pathobiology, Addis Ababa University, Addis Ababa, Ethiopia; University of Michigan, UNITED STATES OF AMERICA

## Abstract

**Background:**

There was a ‘missing millions’ gap between the incidence of tuberculosis (TB) cases and the notified cases. In many TB high-burden countries, only about 25% of household contacts (HHCs) completing household TB evaluation and 20–89% of eligible contacts did not adhere to TB screening. The study was conducted to assess the yield of door-to-door TB household contact investigation among household contact of bacteriologically confirmed pulmonary TB cases in central Ethiopia.

**Methods:**

This cross-sectional study was carried out in selected health facilities of central Ethiopia from January 1, 2023 to December 3, 2023.All sequential voluntary bacteriologically confirmed TB patients and their HHCs without discrimination by age were included in the study. Xpert Ultra assay and TB culture were used to investigate active TB from sputum sample. Spearman’s correlation analysis was used to determine the correlation between the index case cycle threshold value and the corresponding HHCs. Multivariable logistic regression analysis was done to investigate the associated risk factors for active TB in HHCs.

**Results:**

Among 967 HHCs claimed by 303 index cases (259 drug susceptible TB (DS-TB) and 44 multi-drug resistance TB (MDR/RR-TB)), 902(93.07%) HHCs had received baseline symptom-based TB evaluation. Presumptive TB was identified in 20.17% (182) of the evaluated HHCs and 13(1.44%) were diagnosed with active TB. Eleven HHCs (7.24%; 95% CI: 3.85–12.9%) from DS-TB index case contacts and 2 (6.67%; 95% CI: 1.16–23.51) from MDR/RR-TB indexes HHCs were found to be MTB detected Rifampicin resistance not detected cases. The Xpert ultra assay results revealed an 84.62% (95% CI: 57.77–95.68) Rifampicin drug resistance concordance between the index case and the corresponding HHC. Active TB was significantly associated with night sweating and sharing a bed with the index patient, P-value < 0.05.

**Conclusion:**

Home-to-home TB contact screening have high active TB yield and implementable in both rural and urban areas of the nation only by mentoring and motivating the health extension workers. Proximity to bacteriologically confirmed TB patient for long time exposes household contacts for active TB. Scheduling convenient times and last-mile service delivery to contacts is very important to address the missed active TB cases in the community.

## Introduction

Worldwide in 2023, 10.8 million people developed tuberculosis (TB), the disease is estimated to have killed 1.25 million people. It proceeds to be the second most frequent cause of mortality from a single infectious pathogen. According to 2025 WHO end TB milestone, there was a ‘missing millions’ gap between the incidence of TB cases and the notified TB cases. The bulk of missing persons with TB live in low-income countries, and many of them continue to have symptoms without seeking medical attention [1].

Active (TB) refers to a disease that occurs in a person infected with *Mycobacterium tuberculosis* (Mtb) and it occurs when the immune system cannot defend against an infection [2]. Once an individual is newly diagnosed with TB, the most effective procedure for managing household contacts (HHCs) includes screening for TB, treating any individual who has TB disease, and giving TB preventive treatment (TPT) to those with TB infection but not diseased based on eligibility criteria. This improves the early identification and management of contacts with disease. This recommended strategy is active TB contact investigation [3].

The outcome of TB by active case finding is reliant on the screening algorithm, the characteristics of the contacts being evaluated, and most importantly, the linkage between effective diagnostic method and treatment facilities [4]. The routine strategy to contact investigation is that the index patient is requested to declare the number of HHCs and bring all of them to the health facility for symptom evaluation and TPT initiation, if eligible. This passive method is hampered by several factors, including extended waiting time, difficulties with scheduling or finances, and reluctance by parents or health professionals towards screening and commencing TPT in a healthy child [5].

The main predictors of tuberculosis but not limited to are poverty, undernourishment, HIV infection, smoking, and diabetes [1]. The risk of TB among contacts is determined by the characteristics of the index cases and contacts, or the environment setting where the exposure happened. Bacteriological confirmation of TB and cavitary spots in the index patient could facilitate the release of large quantities of bacteria and increase the likelihood of transmission [6]. Many publications have shown an association between the bacillary load in sputum and Mtb transmission [7,8,9]. Close contacts of MDR-TB patients are more likely to contract drug resistance TB (DR-TB). Meanwhile, contradicting evidence has been reported from many studies regarding the risk of TB in close contacts of drug-susceptible and MDR-TB patients [8,9,10].

In many TB high-burden countries contact investigation access and uptake was poor, with only about 25% of household contacts completing household TB evaluation and 20–89% of eligible contacts did not adhere to TB screening [11,12]. The key bottlenecks to the uptake and adherence of contact investigation include a lack of TB-specific awareness; the stigma associated with the disease; catastrophic costs; and dissatisfaction with the quality of health facility general services [13,14].

The most effective way to curve the incidence rate is to reach the most at-risk group. This study closes this gap by bringing TB contact investigation service closer to people in need thereby subsidizing catastrophic costs, delayed diagnosis, and treatment delay. The present study was conducted to study the yield of door-to-door TB household contact investigation among (HHC) of bacteriologically confirmed pulmonary TB cases in central Ethiopia.

## Methods

### Study design and setting

This cross-sectional study was conducted in central Ethiopia from January 1, 2023 to December 3, 2023. It includes a 200-kilometer radius from the capital Addis Ababa. It covers three zones of the Oromia region, the Addis Ababa city administration, one zone from Amhara region, and one zone from the central Ethiopia region. This setting is among the densely populated areas in Ethiopia, with an average of 398.4 persons per km2 and an average family size of 3.1 [15]. In Ethiopia TB care and management is decentralized to primary health care facility which meets prerequisites to give the service. In the study area, 42 public health hospitals and 372 health centers provide TB diagnostic and treatment services [16].

### Tuberculosis case identification and management in Ethiopia

In Ethiopia, hospitals or health centers are the facilities where the first TB diagnosis and treatment initiation take place. After that, patients are referred back to the health posts that are nearest to their place of residence for the remainder of their care. Health extension workers (HEWs) ensure adherence to treatment and follow-up through daily observation. HEW routinely visits households under their catchment area. During their visit, they will identify and refer presumptive TB cases, trace treatment interrupters and lost to follow-up, give health education, and perform household contact screening [17].

### Sample size calculation

The sample size was calculated using a single-population proportion formula with a 5% level of significance and a 1% margin of error.

According to earlier systematic reviews and meta-analyses, at baseline, 2.3% of household contacts screened had TB [18]. Hence, using these figures, the sample size was calculated to be 856, and including a 10% non-response rate the final sample size was 942.

Since it is not feasible to directly sample HHCs of TB patients, index TB cases were selected and all eligible HHCs were enrolled in the study. According to the national TB program, there were 3.1 HHCs per index TB patient. Based on these data we recruited 303 bacteriologically confirmed pulmonary TB patients without discrimination by drug resistance profile.

### Sampling procedure

Initially, a list of all TB diagnostic and treatment health facilities in the study area was obtained from the Ministry of Health. Based on the previous year TB caseload sites were grouped into high, medium, and low. Using a simple random method three sites were selected from each category. In central Ethiopia, there are seven drug-resistant tuberculosis (DR-TB) treatment initiating centers (TICs). We selected five of them randomly. The total sample size was proportionally distributed to each site. All consecutive voluntary bacteriologically confirmed PTB index patients and their HHCs were included in the study.

### Operational definition of variables

An active pulmonary TB case is a TB case confirmed by either smear microscopy, Gene Xpert MTB/RIF Ultra, or a TB culture test. Bacteriologically confirmed pulmonary tuberculosis: case referred to a pulmonary TB patient with biological specimen positive by Acid-Fast Bacilli (AFB) smear microscopy, Xpert MTB/RIF assay or TB culture, indifference with drug susceptibility profile. A household contact was defined as a person who shared the same enclosed living space as the index case for one or more nights or for frequent or extended daytime periods during the three months before the start of TB treatment. An index case was the first bacteriologically confirmed TB case in a household at any age that lived with at least one other person.

### Study participants

Every consented consecutive bacteriologically confirmed pulmonary TB patient (index cases) who had visited TB clinics at any age, had given a traceable residence location, and had one or more household contacts was eligible for the study. The study included index cases without discriminating by drug resistance profile. The study did not include prison index cases due to restricted entry.

As long as a HHC had lived with the infectious TB index case for at least three months, had not received TB treatment at or before the time of the home visit, had given their consent to the study, and was available for interviews during the home visits; they were included in the study regardless of age.

### Contact investigation procedure

This study executed a nationally recommended active TB contact investigation strategy for the evaluation and management of HHCs at the dwelling. Once an individual is diagnosed with bacteriologically confirmed pulmonary TB (index case), the health personnel in the TB clinic request the index case to list all household contacts that live with him in the same dwelling. The guardians or parents of children index case, under 18 years, were asked for their consent. If two or more index cases were not diagnosed on the same day; one index case means one household. The head of the household was asked whether s/he is voluntary to host HEW in his/her dwelling for contact TB symptom evaluation. The HEW visits the household in the morning section of the scheduled date.

On the booked date the HEWs asked verbal permission to give health education related to TB and conduct TB symptom screening. When HHCs missed the first visit second round appointment was made to address them. If they missed the second time they were counted as missing the evaluation. Household contacts having contact with the index case were examined for active TB through symptom screening. A presumptive TB case was a contact with cough of two weeks or more or having any two of the following symptoms: fever of two weeks or more, night sweats, and unexplained weight loss of more than 1.5 kg in a month. Index cases were requested to give sputum samples before they started TB treatment. Tuberculosis symptomatic HHCs gave early morning sputum for bacteriological confirmation of active tuberculosis. Those who were unable to produce appropriate samples were advised to try during the second round of home visits. Tuberculosis-asymptomatic HHCs were advised to come to health posts or health facilities whenever they experience TB symptoms (S1 Fig).

### Microbiological evaluation for active TB cases

The collected samples were transported to the Ethiopian Public Health Institute using cold chain courage (2–8^o^c) through the postal system within the collection date. The samples were examined for TB using Xpert Ultra assay, smear microscopy, and Mtb culture. The result was communicated to the health facility clinician who sent the sample immediately after the release of the result. The principal investigator and the clinician followed the initiation of TB treatment for active TB-positive household contacts. Under 15 years of children (contacts of drug-susceptible index case) who ruled out TB were linked to TPT.

All specimens were processed using the NALC-NaOH (N-acetyl L-cystine sodium hydroxide sodium citrate) digestion decontamination technique described in the Global Laboratory Initiative (GLI) manual (19). Following processing, 0.5 ml was added to liquid media Mycobacteria Growth indicator tube (MGIT) and 0.1 ml inoculated to LJ(Lowenstein Jenson) media, AFB(Acid fast bacilli) smear was prepared and stained with the Zehil Nelsson (ZN)Staining method. Tuberculosis culture is highly sensitive with lower detection limit of 10–100 CFU/ml. Xpert Ultra assay was done within 24 hours after the arrival of the samples at the reference laboratory. Xpert Ultra assay has a sensitivity of 90% (95%CI: 84–94) and specificity 96%(95%CI:93–98).The result interpretation and test procedure were done based on the test user manual guide [19,20].

### Data collection and quality assurance

A data collection tool was developed to collect socio-demographic characteristics, clinical findings, risk factors for active TB, and laboratory results. The data collection tool was evaluated using 5% of the sample size. The lead investigator verified the accuracy and completeness of the data every day. Starting and ending controls were included in every batch of MTB culturing. The collected data were analyzed and interpreted accordingly after it was checked for completeness, accuracy, and clarity. The sterility of the culture media, sample processing reagents, and performance of the media were checked by incubating the whole media at 37 ^0^C for 48 hours, inoculating all reagents in a separate BHI and known susceptible *M*. *tuberculosis* (H37Rv), respectively. All laboratory results were recorded in a logbook and transformed into the data collection tool.

### Data analysis

The data was cleaned and entered in Epi data version 4.6 and exported to STATA version 17 software for analysis. The yield of contact investigation at each cascade was depicted using a processed map. Descriptive statistics were used to summarize data. Spear’s man correlation analysis was done to determine the correlation between the index case ct value and the respective HHC. Binary logistic regression analysis was done at two levels. Groups were compared using a Chi-square test with Yates correction of continuity. Variables with P-values of 0.25 in bivariate logistic regression analysis were moved into multivariable logistic regression analysis. Finally, the adjusted odds ratio (aOR) with 95% confidence intervals and P-value < 0.05 was considered as statistically significant.

### Ethical consideration

The study was approved by Addis Ababa University, Aklilu Lema Institute of Pathobiology, with approval number ALIP IRERC/94/2015/23 and Ethiopian Public health Institute, EPHI-IRB-456-2022. The Addis Ababa Health Bureau also provided an official letter of support for the study. Following oral and written explanations of the study, voluntary index patients, HHCs, and guardians (when the index case or the HHC was children aged less than 15 years) signed written informed consent and assent. Individual records were closely protected in confidence, and data anonymization was ensured by aggregate analysis. Treatment of bacteriologically confirmed active TB patients (HHCs) and administration of TPT to children fifteen years of age or under who do not have active TB were conducted by the national TB guidelines.

## Result

**Fig 1:**
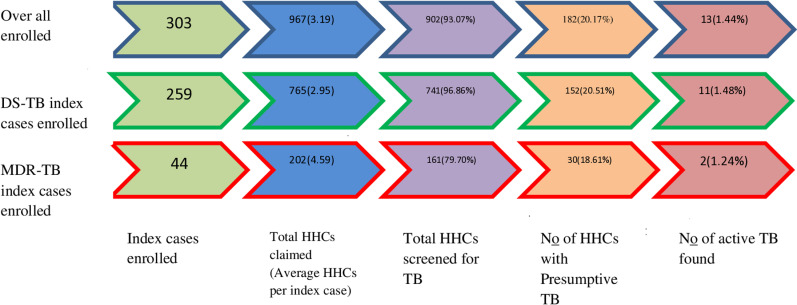
Home –to-home TB contact investigation cascade in central Ethiopia from January 1 to December 3, 2023.

A total of 335 bacteriologically confirmed PTB index patients were reached within the study period, and 303 of them were enrolled by fulfilling the study’s inclusion criteria. The main reason index patients were ineligible was that they declared no HHC. Of the included index patients, 259 were DS-TB patients, while 44 were MDR-TB patients. A total of 967 HHCs were reported by the included index patients. The average number of HHCs per index case was 3.19(range 1–7). MDR-TB index cases had more HHCs per index case than DS-TB index cases (4.59 and 2.95, respectively) (Fig 1).

During household visits, a total of 902(93.07%) HHCs had received baseline symptom-based TB evaluation. The proportion of HHCs screened among the declared total HHCs was lower in contacts of MDR-TB index cases than in DS-TB index cases (79.70% and 96.86%, respectively). Presumptive tuberculosis was identified in 182 (20.17%) of the evaluated HHCs. All presumptive HHcs produced sputum samples for TB laboratory diagnosis. In the DS-TB and MDR-TB index case groups, 11 (1.48%) of 765 HHcs and 2 (1.24%) of 161 HHCs were diagnosed with active tuberculosis, respectively. All the 13 active tuberculosis cases found from HHCs were culture positive. This gave 1.44% TB prevalence among HHCs enrolled in the study (Fig 1).

The median age and BMI of TB symptom-screened HHCs were 28 years and 20.57, respectively. Among the screened HHCs, 513(56.83%) were female, 92(10.20%) were under 15 years children, and 167(18.51%) were under weight. The most prevalent symptom was a cough of any duration, 214 (23.73%). According to the national TB case management guideline, only 182 (20.17%) HHCs had presumptive TB. The most common symptom among TB symptom screening positive HHCs was cough with any duration 214 (23.73%) followed by fever with any duration 79 (8.76%). Among household contacts of index patients, 167(18.51%) were underweight, 206(22.89%) had BCG vaccination scars on their hands and 92 (10.20%) had comorbid disease. Only, 43 (4.77%) HHCs reported that they either currently smoke or had a history of smoking cigarettes (Table 1).

**Table 1:** Characterstics of bacteriologically confirmed pulmonary index cases and their TB screened HHCs in central Ethiopia from January 1 to December 30, 2023.

Characteristics		Index case; N(%) = 303	HHCs; N (%) = 902
Sex	Male	168(55.45)	389(43.17)
	Female	135 (44.55)	513(56.83)
Age	Median		28(20–38)
	<15	7 (2.31)	92(10.20)
	16–24	70 (23.10)	248(27.49)
	25–34	114 (37.62)	269(29.82)
	35–44	60 (19.80)	144 (15.96)
	45–65	40(13.20)	139 (15.41)
	>65	12 (3.96)	10 (1.11)
BMI	Under weight(<18.5)	NA	167(18.51)
	Normal weight(18.5–24.9)	NA	709(78.60)
	Over weight(>25.0)	NA	26 (2.88)
Educational status	Illiterate	36(11.88)	54 (5.99)
	Read and write	15(4.95)	26 (2.89)
	Primary	70 (23.10)	155 (17.20)
	Secondary	97 (32.01)	354 (39.18)
	Certificate and above	85 (28.05)	313 (34.74)
Route transmission know	Yes	NA	591 (65.52)
	No	NA	311 (34.48)
Relation with Index	Husband	NA	80 (8.88)
	Wife	NA	64 (7.10)
	Child	NA	242 (26.75)
	Other	NA	233 (25.86)
	Relative	NA	283 (31.41)
BCG vaccinated(have scar)	Yes	NA	206(22.89)
	No	NA	696 (77.11)
cough > 2 weeks	Yes	NA	182(20.17%)
	No	NA	720(79.83%)
Cough any duration	Yes	NA	214 (23.73)
	No	NA	688 (76.27)
Fever any duration	Yes	NA	79 (8.76)
	No	NA	823 (91.24)
Weight loss	Yes	NA	26 (2.88)
	No	NA	876 (97.12)
Night sweeting	Yes	NA	32 (3.55)
	No	NA	870 (96.45)
Chest pain	Yes	NA	9 (1.00)
	No	NA	893 (99.00)
HIV status	Positive	45 (14.85)	21 (2.33)
	Negative	255 (84.16)	388 (43.02)
	Unknown	3 (0.99)	493(54.66)
Have underline medical condition	Yes	NA	92 (10.20)
	No	NA	810 (89.80)
Time with Index	All time	NA	507(56.21)
	Night	NA	256 (28.38)
	Day	NA	129 (14.30)
	Other	NA	10 (1.11)
Drink alcohol daily	Yes	44 (14.52)	120 (13.30)
	No	259 (85.48	782 (86.70)
History/Current smoker	Yes	23 (7.59)	43 (4.77)
	No	280 (92.41)	859 (95.23)
Share bed	Yes	NA	208 (23.06)
	No	NA	694 (76.94)

NA=Not applicable/no data.

**Fig 2:**
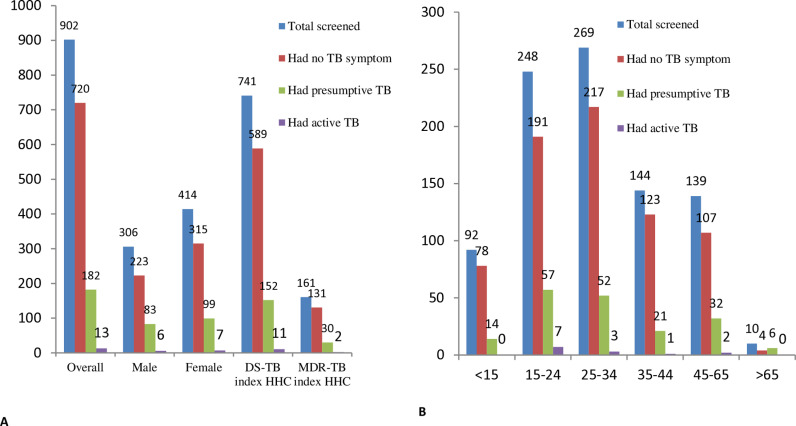
Proportion of Asymptomatic, presumptive TB and active TB in HHCs: by A. sex, index Drug resistance type and overall B. age group in central Ethiopia from January 1 to December 3, 2023.

The proportion of total TB symptom screened HHCs among declared, presumptive TB, bacteriologically confirmed active pulmonary TB cases diagnosed varied by sex, age, and index drug susceptibility type. There were overall differences in the proportions of presumptive TB cases by age groups(<15 years old,15.21%;15–24 years,27.12%;25–34 years,19.33%;35–44 years,14.58%;45–65 years,23.02% and > 65 years,60.0%;p-value = 0.007). Even though it was not statistically significant (p-value = 0.45) presumptive TB cases differ by sex (27.12% in males and 23.91% in females). The proportions of bacteriologically confirmed active TB cases by age groups were (<15 years old,0.00%;15–24 years,2.82%;25–34 years,1.11%;35–44 years,0.69%;45–65 years,1.43% and > 65 years,0.00%;p-value = 0.34) (Fig 2).

The following risk factors were found to associate with risk of presumptive TB after adjustment for these potential confounders: age > 65 (aOR(95%CI), 15.15(2.44–94.11), illiterate educational status (aOR(95%CI), 2.41(1.08–5.93), relation with index other than parents (aOR(95%CI), 0.50(0.28–0.91),having underline medical condition(aOR(95%CI),1.99(1.08–3.66), night time exposure to index case (aOR(95%CI),0.33(0.22–0.49) day time exposure to index case(aOR (95%CI), 0.44(0.22–0.86) (Table 2). Active TB positivity was significantly associated with night sweating, chest pain, any duration of fever, weight loss, night sweat and sharing a bed with the index patient, P-value < 0.05 (Table 3).

**Table 2:** Comparative characteristics of tuberculosis symptom screening positive and screening negative household contacts at base line visit in central Ethiopia from January 1 to December 30, 2023.

Variable		Symptomatic household contacts (n = 182(20.17%))	Asymptomatic household contacts (n = 740(79.83%))	AOR(95%CI)	P-value
Sex	Female	99(19.30)	414 (80.70)	1	
	Male	83(21.34)	306(78.66)	1.13(0.74–1.71)	0.55
Age	<15	14 (15.22)	78 (84.78)	1	
	16–24	57 (22.98)	191(77.02)	2.29(0.96–5.45)	0.06
	25–34	52 (19.33)	217 (80.67)	2.55(0.90–7.21)	0.07
	35–44	21 (14.58)	123 (85.42)	1.53(0.49–4.72)	0.45
	45–65	32 (23.02)	107 (76.98)	2.64(0.89–7.76)	0.07
	>65	6 (60.00)	4 (40.00)	15.15(2.44–94.11)	**0.01**
BMI	Under weight(<18.5)	40 (23.95)	127 (76.05)	1	
	Normal weight(18.5–24.9)	139 (19.61)	570 (80.39)	0.70(0.42–1.17)	0.18
	Over weight(>25.0)	3 (11.54)	23 (88.46)	0.47(0.11–1.97)	0.30
Educational status	Primary	29(18.71)	126 (81.29)	1	
	Illiterate	16(29.63)	38 (70.37)	2.41(1.08–5.93)	**0.05**
	Read and write	8 (30.77)	18 (69.23)	2.31(0.76–6.95)	0.13
	Secondary	66 (18.64)	288 (81.36)	0.82(0.44–1.50)	0.52
	Certificate and above	63 (20.13)	250 (79.87)	0.90(0.45–1.80)	0.78
Route of transmission know	Yes	127(21.49)	464 (78.51)	1.30(0.83–2.02)	0.23
	No	55 (17.68)	256 (82.32)	1	
Relation with Index	Child	52 (21.49)	190 (78.51)	1	
	Husband	19 (23.75)	61 (76.25)	0.76(0.34–1.69)	0.51
	Wife	21 (32.81)	43 (67.19)	1.82(0.83–3.97)	0.13
	Other	27 (11.59)	206 (88.41)	0.50(0.28–0.91)	**0.02**
	Relative	63 (22.26)	220 (77.74)	1.14(0.70–1.85)	0.59
BCG vaccinated	Yes	47 (22.82)	159 (77.18)	1.06(0.60–1.86)	0.83
	No	135(19.42)	560 (80.58)	1	
HIV status	Negative	115(29.64)	273(70.36)	1	
	Positive	10 (47.62)	11 (52.38)	1.45(0.51–4.10)	
	Unknown	57 (11.56)	436 (88.44)	0.33(0.22–0.49)	**0.01**
Underline medical condition	Yes	31 (33.70)	61 (66.30)	1.99(1.08–3.66)	**0.02**
	No	151(18.64)	659 (81.36)	1	
Time with Index	All time	120 (23.67)	387 (76.33)	1	
	Night	46(17.97)	210 (82.03)	0.64(0.41–0.99)	**0.04**
	Day	13 (10.08)	116 (89.92)	0.44(0.22–0.86)	**0.01**
	Other	3 (30.00)	7 (70.00)	1.25(0.27–5.67)	0.77
Drink alcohol daily	Yes	25 (20.83)	95 (79.17)	0.83(0.43–1.59)	0.58
	No	157 (20.08)	625 (79.92)	1	
Current smoker	Yes	13 (30.23)	30 (69.77)	2.20(0.89–5.39)	0.08
	No	169 (19.67	690 (80.33)	1	
Share bed	Yes	50 (24.04))	158 (75.96)	1.05(0.65–1.70)	0.82
	No	132 (19.02)	562 (80.98)	1	

**Table 3:** Risk factors for active tuberculosis in household contacts of bacteriologically confirmed pulmonary tuberculosis patients in central Ethiopia from January 1 to December 30, 2023.

variable		Active TB diagnosed (%)	Without active TB (%)	Chi-Square test	P-value*
Total		13/902(1.44%)	889/902(98.56)		
HHCs sex	Male	6 (1.54)	383 (98.46)	0.61	0.61
	Female	7 (1.36)	506 (98.64)		
Route of transmission know	Yes	9 (1.52)	582 (98.48)	0.08	0.99
	No	4 (1.29)	307 (98.71)		
BCG vaccinated	Yes	5 (2.43)	201 (97.57)	0.01	0.99
	No	8 (1.15)	687 (98.85)		
Any duration fever	Yes	3 (3.80)	76 (96.20)	14.56	<0.001
	No	10 (1.22)	813 (98.78)		
Weight loss	Yes	3 (11.54)	23 (88.46)	36.64	<0.001
	No	10 (1.14)	866 (11.54)		
Night sweat	Yes	4 (12.50)	28 (87.50)	69.97	<0.001
	No	9 (1.03)	861 (98.97)		
Chest pain	Yes	2 (22.22)	7 (77.78)	27.63	<0.001
	No	11 (1.23)	882 (22.22)		
Have underline medical condition	Yes	2 (2.17)	90 (97.83)	0.38	0.87
	No	11 (1.36)	799 (98.64)		
Drink alcohol daily	Yes	1 (0.83)	119 (99.17)	0.05	0.99
	No	12 (1.53)	770 (98.47)		
History/Current smoker	Yes	1 (2.33)	42 (97.67)	3.27	0.24
	No	12 (1.40)	847 (98.60)		
Share bed	Yes	7 (3.37)	201 (96.63)	3.96	0.05
	No	6 (0.86)	688 (99.14)		

*Yates continuity correction P-value.

**Fig 3:**
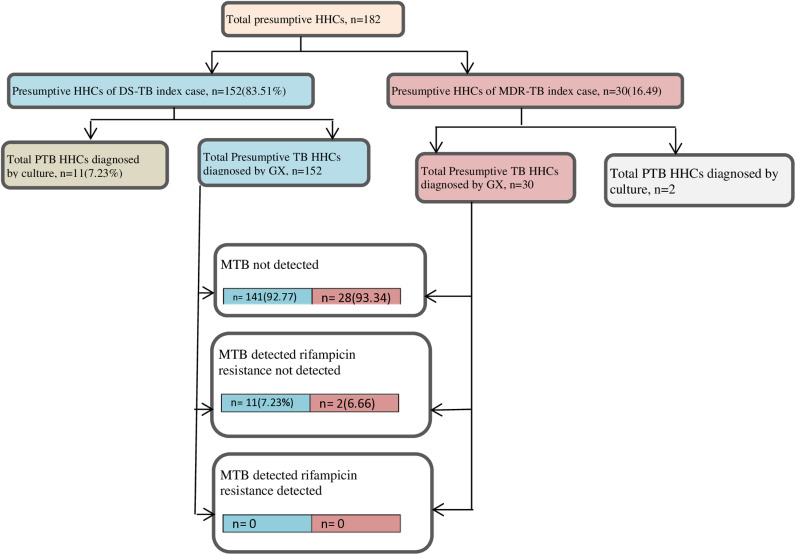
Bacteriological confirmation of household contacts having active pulmonary TB from January 1 to December 3, 2023. Purple color is for MDR-TB index case and Blue color is for DS-TB index case.

Among the DS-TB index case contacts, 152 (20.51%; 95% CI: 17.76–23.57) were diagnosed with presumptive TB, and from these 11 (7.24%; 95% CI: 3.85–12.9%) were found to be MTB detected Rifampin resistance not detected cases by Xpert Ultra assay and culture test. Whereas among thirty presumptive household contacts of MDR-TB patients, 2 (6.67%; 95% CI: 1.16–23.51) were MTB detected Rifampicin resistance was not detected. Rifampicin resistance active PTB was not detected in both study groups. The Xpert ultra assay results revealed an 84.62% (95% CI: 57.77–95.68) rifampicin drug resistance concordance between the index case and the corresponding household contact (Fig 3).

**Fig 4:**
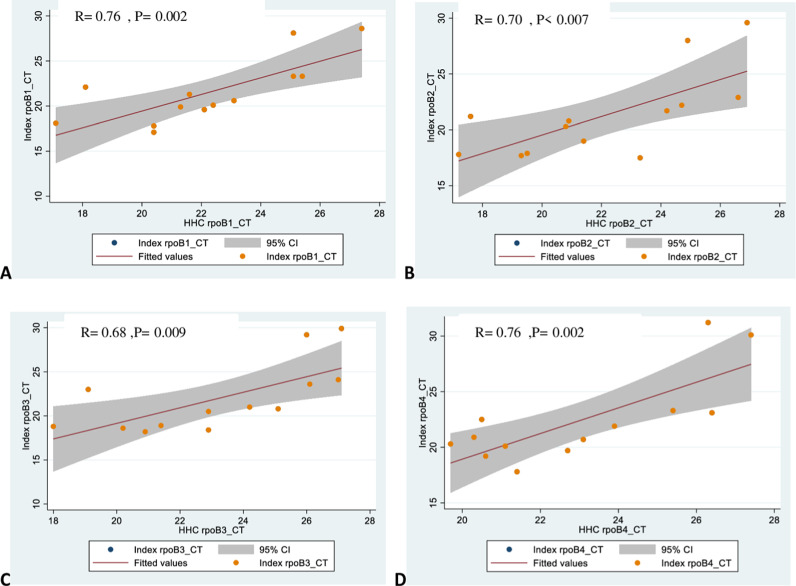
Spearman correlation between Xpert CT parameters of index cases and HHCs. (A). Xpert CT rpoB1; (B). Xpert CT rpoB2; (C). Xpert CT rpoB3 and (D). Xpert CT rpoB4.

A Spearman correlation analysis was performed to determine the relationship between the Xpert MTB/RIF probe cycle thresh hold (ct) values of the index case and their respective HHCs. There was a positive correlation between the ct values of the index case and the corresponding HHC ct value at each probe. This analysis indicates a substantial correlation between the ct values of the index cases and the corresponding HHC, indicating that there is an association between the TB index ct values and corresponding HHc ct values that might be related to TB transmission. There is an increased risk of TB transmission to close contacts when the TB index case has a high bacillary load (Fig 4).

## Discussion

Household contacts of patients with bacteriologically confirmed pulmonary tuberculosis are significantly at higher risk of contracting active tuberculosis. To curve tuberculosis trend under any setting, evaluation of these household contacts under any setting should be a crucial component of the public health response. This study found that home-to-home TB contact investigation had a higher active TB yield than the routine TB contact investigation implemented in similar health facilities [21]. This indicates that scheduling convenient times and last-mile service delivery to contacts is very important to address the missed active TB cases in the community. To the best of our knowledge, this is the first door-to-door active case finding of all age group household contacts of patients with bacteriologically confirmed DS-TB or MDR-TB in central Ethiopia. Using home-to-home active TB case screening in central Ethiopia, we found, on average, 144 HHCs with active TB per 10000 household contacts screened. We noticed that booking a convenient time (at least two times, one is for those who missed the first visit) for all HHC members increases screening adherence and therefore yields active TB.

Our study reported a yield of 1.44% active TB amongst HHCs of bacteriologically confirmed pulmonary TB index cases that were screened using the Ministry of Health-approved symptom screening method only. Previous studies have reported different proportions of active tuberculosis from HHC investigations. Our result is similar to that documented in a study conducted in Nepal, Guniea, and China which reported 1.6% [22], 1.6% [23], and, 1.7% [24], respectively. It was higher than the reports from Vietnam (1.0%) [25], India (1.15%) [26], Iran (1.1%) [27], and in Ethiopia (1.1%) [18]. However, it was lower than the community TB screening finding from Uganda which reported 2.35% [28], and from Indonesia 2.4% [29]. This variation might be attributed to differences in the study populations, and study settings including TB burden, household ventilation, and sleeping arrangements, community living habits, health-seeking behavior of household contacts, infectiousness of index cases, vulnerability of contacts, and study methodology, with differences in sample size, screening algorithm, and diagnostic accuracy. The screening algorithm (only symptomatic HHC were privileged to give sputum samples without support by chest x-ray) used in our investigation might affect the yield of active tuberculosis.

Although the number of MDR-TB index patients in our study was fewer than the number of DS-TB index cases, we found no significant difference in active tuberculosis contact investigation yield among HHCs based on the drug resistance profile of the index case. A similar study finding was reported from Ethiopia [30]. MDRTB patients are less likely to infect others than patients with drug-susceptible tuberculosis [31]. This makes our finding welcome but it lacked statistical power to distinguish between the number of secondary cases in MDR-TB index cases HHCs versus DS-TB index case contacts.

The yield of HHCs investigation is impacted by several factors, and it deteriorates when it comes to children’s contact. In this study, there was a significant prevalence of presumptive TB in children (aged less than 15 years), but no active TB cases were detected. Our study findings were in contradiction with studies from Tajikistan [32] Myanmar [33] and Pakistan [34] which reported active TB cases from children’s household contacts. In our study, the majority of the index cases were not parents; children were more proxies for their parents than other family members, potentially affecting the yield of active tuberculosis in children. Even though we were capable of collecting samples from all presumptive cases, the study’s sample-collecting strategy did not enhance the production of suitable samples from children. It suggests the need for additional techniques to take appropriate samples from children. After tuberculosis was ruled out, preventive therapy was commenced for all children based on the eligibility criteria.

In our study, we found a high uptake for TB symptom screening (93.07%) and testing for active TB among HHCs. A similar finding was reported from South Africa(95% screened at baseline) [35]. Engaging full-time, salaried, and familiar with the community HEWs in TB active-case finding schemes might help us to achieve much-appreciated achievement in this target. To get high symptom screening uptake scheduling a convenient time when most of the HHCs are available is important MDR-TB index case’s HHcs had lower TB symptom screening than DS-TB index case’s HHCs. This might be the fact that relatively DOT facilities were closer to patients’ residences than MDR-TB TICs. Around one–fifth of the HHCs were symptomatic for PTB, this result was lower than the finding from Myanmar (39%) [33] but it was higher than the reports from Ethiopia (13%) [18] Delivery of TB screening to the door of HHCs might increase the need of the contacts to be tested which forced them to falsely report the symptoms.

The risk factors (sharing a bed with an index patient and night sweetening) for active tuberculosis revealed in this study were comparable to those from the Chinese study [29,36]. The risk of contracting TB from an infectious patient increases as the proximity and duration of exposure to the index case increases [37]. Even though the exact cause of night sweating is still unknown, it is a vital sign that should not be disregarded while screening for tuberculosis [38].

The Xpert MTB/RIF Ultra assay results from this study indicate there was a high concordance of rifampicin drug resistance (84.62%) between the index case and the corresponding household contact. This finding was similar to the result of the meta-analysis pooled estimate [39] which had reported the pooled Pooled isoniazid/rifampicin concordance was 82.6% and report from Pakistan [10]. In a single household, it is possible for two individuals to have been exposed to different M. tuberculosis strains from the surrounding community. Studies have described that a significant number of contacts living in the same household possess different drug-resistance profiles in contrast to the index patients [10,39]. However, it must be supported by a whole genome sequencing test.

The significant strength of the study was its pragmatic nature, which employed the existing system established by the national tuberculosis program, healthcare professionals work in the health facility and HEWs work in the community. This indicates that home-to-home TB contact screening was effective and implementable in both rural and urban areas of the nation only by mentoring and motivating the health extension workers. To ensure early detection and treatment of TB, healthcare providers must screen HHCs of patients with bacteriologically confirmed TB in timely manner.

One of the limitations of our study is the possibility of over-diagnosis which results from HHCs falsely reporting TB symptoms by considering as a great chance of being diagnosed at home. We did not use clinical screening like chest X-rays which might increase the sensitivity of screening. Additionally, we were unable to screen all the listed household contacts which may have resulted in the estimation of active TB yield. Financial and resource shortages limited us to testing latent TB infection in the HHCs. We suggest conducting additional research using whole genome sequencing to identify the actual index case and understand the transmission dynamics.

## Conclusion

Home-to-home TB contact screening have high active TB yield and implementable in both rural and urban areas of the nation only by mentoring and motivating the health extension workers. Proximity to bacteriologically confirmed TB patient for long time exposes household contacts for active TB. Incorporating door-to-door TB investigations into existing public health structures, such as utilizing health extension workers and women’s community groups, could improve TB control strategies. Scheduling convenient times and last-mile service delivery to contacts is very important to address the missed active TB cases in the community. To combat this catastrophic infectious disease we have to strength our strategy and leave the reluctance.

## Supporting information

S1 Fig Tuberculosis screening algorithm for HHCs. (DOCX)

## Abbreviations

AOR: Adjusted Odds Ratio
CI: Confidence Interval
HEW: Health Extension Workers
HHC: Household contacts
PTB: Pulmonary Tuberculosis
TB: Tuberculosis
WHO: World Health Organization.

## References

[1] Global tuberculosis report 2024. Geneva: World Health Organization; 2023. Licence: CC BY-NC-SA 3.0 IGO.

[2] World Health Organization. Systematic screening for active tuberculosis: principles and recommendations [Internet]. Geneva: WHO; 2013. [Last cited on 2024 Jun 06]. 25996015

[3] WHO. WHO consolidated guidelines on tuberculosis: module 5: management of tuberculosis in children and adolescents. 2022. 35404556

[4] Prathiksha G, Daniel BD, Natrajan M. Active case-finding for tuberculosis in India. Natl Med J India. 2019;32(2):90–5. doi: 10.4103/0970-258X.275349 31939405

[5] Vasiliu A, Tiendrebeogo G, Awolu MM, Akatukwasa C, Tchakounte BY, Ssekyanzi B, et al; CONTACT study group. Feasibility of a randomized clinical trial evaluating a community intervention for household tuberculosis child contact management in Cameroon and Uganda. Pilot Feasibility Stud. 2022 Feb 11;8(1):39. doi: 10.1186/s40814-022-00996-3 35148800 PMC8832743

[6] Pinto PFPS, Teixeira CSS, Ichihara MY, Rasella D, Nery JS, Sena SOL, et al. Incidence and risk factors of tuberculosis among 420 854 household contacts of patients with tuberculosis in the 100 Million Brazilian Cohort (2004-18): a cohort study. Lancet Infect Dis. 2024 Jan;24(1):46–5656. doi: 10.1016/S1473-3099(23)00371-7 37591301 PMC10733584

[7] Najjingo I, Muttamba W, Kirenga BJ, Nalunjogi J, Bakesiima R, Olweny F, et al. Comparison of GeneXpert cycle threshold values with smear microscopy and culture as a measure of mycobacterial burden in five regional referral hospitals of Uganda- A cross-sectional study. PLoS One. 2019 May 15;14(5):e0216901. doi: 10.1371/journal.pone.0216901 31091275 PMC6519814

[8] Melsew YA, Doan TN, Gambhir M, Cheng AC, McBryde E, Trauer JM. Risk fac tors for infectiousness of patients with tuberculosis: a systematic review and meta- analysis. Epidemiol Infect. 2018;146(3):345–53. doi: 10.1017/S0950268817003041 29338805 PMC9134570

[9] Lange B, Khan P, Kalmambetova G, Al-Darraji HA, Alland D, Antonenka U, et al. Diagnostic accuracy of the Xpert® MTB/RIF cycle threshold level to predict smear positivity: a meta-analysis. Int J Tuberc Lung Dis. 2017 May 1;21(5):493–502502. doi: 10.5588/ijtld.16.0702 28399963

[10] Javaid A, Khan MA, Khan MA, Mehreen S, Basit A, Khan RA, et al. Screening outcomes of household contacts of multidrug-resistant tuberculosis patients in Peshawar, Pakistan. Asian Pac J Trop Med. 2016 Sep;9(9):909–12. doi: 10.1016/j.apjtm.2016.07.017 27633308

[11] Blok L, Sahu S, Creswell J, Alba S, Stevens R, Bakker MI, et al. Comparative meta-analysis of tuberculosis contact investigation interventions in eleven high burden countries. PLoS One. 2015 Mar 26;10(3):e0119822. doi: 10.1371/journal.pone.0119822 25812013 PMC4374904

[12] Armstrong-Hough M, Turimumahoro P, Meyer AJ, Ochom E, Babirye D, Ayakaka I, et al. Drop-out from the tuberculosis contact investigation cascade in a routine public health setting in urban Uganda: a prospective, multi-center study. PLoS One. 2017 Nov 6;12((11):e0187145. doi: 10.1371/journal.pone.0187145 29108007 PMC5673209

[13] Fox GJ, Loan le P, Nhung NV, Loi NT, Sy DN, Britton WJ, et al. Barriers to adherence with tuberculosis contact investigation in six provinces of Vietnam: a nested case-control study. BMC Infect Dis. 2015 Feb 26;15:103. 25886411 10.1186/s12879-015-0816-0 PMC4377211

[14] Ayakaka I, Ackerman S, Ggita JM, Kajubi P, Dowdy D, Haberer JE, et al. Identifying barriers to and facilitators of tuberculosis contact investigation in Kampala, Uganda: a behavioral approach. Implement Sci. 2017 Mar 9;12(1):33. doi: 10.1186/s13012-017-0561-4 28274245 PMC5343292

[15] Ethiopian Public Health Institute (EPHI) and ICF. Ethiopia mini demographic and health survey 2019: final report. Rockville, Maryland, USA: EPHI and ICF. 2021.

[16] Ethiopian Ministry of Health. National tuberculosis and leprosy annual report. Addis Ababa. 2023.

[17] Ministry of Health of Ethiopia. Community Based TB Care Implementation Guidelines 2nd edition - Amharic version, 2013.

[18] Wolde HM, Zerihun B, Sinshaw W, Yewhalaw D, Abebe G. Comparison of the yield of two tuberculosis screening approaches among household contacts in a community setting of Silti Zone, Central Ethiopia: a prospective cohort study. BMC Pulm Med. 2024 Mar 15;24(1):135. doi: 10.1186/s12890-024-02950-w 38491509 PMC10943764

[19] Global laboratory initiative advancing TB diagnostics, mycobacteriology laboratory manual. 2014:1–154.

[20] WHO. Xpert MTB/RIF implementation manual. Geneva. 2014. 25473699

[21] Seid G, Alemu A, Diriba G, Zerihun B, Abebaw Y, Moga S, et al. Routine tuberculosis contact investigation yield and preventive treatment cascade in central Ethiopia. Heliyon. 2024 May 9;10(10):e30942. doi: 10.1016/j.heliyon.2024.e30942 38770348 PMC11103515

[22] Gyawali N, Gurung R, Poudyal N, Amatya R, Niraula SR, Jha P, et al. Prevalence of tuberculosis in household contacts of sputum smears positive cases and associated demographic risk factors. Nepal Med Coll J. 2012 Dec;14(4):303–7. 24579539

[23] Hassane-Harouna S, Gils T, Decroo T, Ortuño-Gutiérrez N, Delamou A, Cherif GF, et al. Community-supported self-administered tuberculosis treatment combined with active tuberculosis screening: a pilot experience in Conakry, Guinea. Glob Health Action. 2023 Dec 31;16(1):2262134. doi: 10.1080/16549716.2023.2262134 37799061 PMC10561566

[24] Leung EC, Leung CC, Kam KM, Yew WW, Chang KC, Leung WM, et al. Transmission of multidrug-resistant and extensively drug-resistant tuberculosis in a metropolitan city. Eur Respir J. 2013 Apr;41(4):901–8. doi: 10.1183/09031936.00071212 22878878

[25] Mac TH, Phan TH, Nguyen VV, Dong TTT, Le HV, Nguyen QD, et al. Optimizing active tuberculosis case finding: evaluating the impact of community referral for chest X-ray screening and Xpert testing on case notifications in two cities in Viet Nam. Trop Med Infect Dis. 2020 Nov 30;5(4):181. doi: 10.3390/tropicalmed5040181 33265972 PMC7709663

[26] Gupta M, Saibannavar AA, Kumar V. Household symptomatic contact screening of newly diagnosed sputum smears positive tuberculosis patients - An effective case detection tool. Lung India. 2016 Mar;33(2):159–62. doi: 10.4103/0970-2113.177445 27051103 PMC4797434

[27] Ghanaiee RM, Karimi A, Hoseini-Alfatemi SM, Seddon JA, Nasehi M, Tabarsi P, et al. Household contact investigation for the detection of active tuberculosis and latent tuberculosis: a comprehensive evaluation in two high-burden provinces in Iran. New Microbes New Infect. 2022 Jan 17;45:100958. doi: 10.1016/j.nmni.2022.100958 35242336 PMC8861284

[28] Turyahabwe S, Bamuloba M, Mugenyi L, Amanya G, Byaruhanga R, Imoko JF, et al. Community tuberculosis screening, testing and care, Uganda. Bull World Health Organ. 2024 Jun 1;102(6):400–9. doi: 10.2471/BLT.23.290641 38812802 PMC11132162

[29] Nababan B, Triasih R, Chan G, Dwihardiani B, Hidayat A, Dewi SC, et al. The yield of active tuberculosis disease and latent tuberculosis infection in tuberculosis household contacts investigated using chest X-ray in Yogyakarta Province, Indonesia. Trop Med Infect Dis. 2024 Jan 31;9(2):34. doi: 10.3390/tropicalmed9020034 38393123 PMC10891579

[30] Hiruy N, Melese M, Habte D, Jerene D, Gashu Z, Alem G, et al. Comparison of the yield of tuberculosis among contacts of multidrug-resistant and drug-sensitive tuberculosis patients in Ethiopia using GeneXpert as a primary diagnostic test. Int J Infect Dis. 2018 Jun;71:4–8. doi: 10.1016/j.ijid.2018.03.011 29559367

[31] Grandjean L, Gilman RH, Martin L, Soto E, Castro B, Lopez S, et al. Transmission of multidrug-resistant and drug-susceptible tuberculosis within households: a prospective cohort study. PLoS Med. 2015 Jun 23;12(6):e1001843; discussion e1001843. doi: 10.1371/journal.pmed.1001843 26103620 PMC4477882

[32] Rekart ML, Aung A, Cullip T, Mulanda W, Mun L, Pirmahmadzoda B, et al. Household drug-resistant TB contact tracing in Tajikistan. Int J Tuberc Lung Dis. 2023 Oct 1;27(10):748–53. doi: 10.5588/ijtld.23.0066 37749832 PMC10519379

[33] Kyaw NTT, Sithu A, Satyanarayana S, Kumar AMV, Thein S, Thi AM, et al. Outcomes of community-based systematic screening of household contacts of patients with multidrug-resistant tuberculosis in Myanmar. Trop Med Infect Dis. 2019 Dec 25;5(1):2. doi: 10.3390/tropicalmed5010002 31881646 PMC7157714

[34] Jaswal MR, Farooq S, Hussain H, Shah J, Nasir K, Khalil A, et al. TB disease yield from household contact screening of tuberculosis index patients; a cohort study from Karachi, Pakistan. TB Outbreaks Week. 2023 May 9;437. *Gale OneFile.*

[35] Martinson NA, Lebina L, Webb EL, Ratsela A, Varavia E, Kinghorn A, et al. Household contact tracing with intensified tuberculosis and human immunodeficiency virus screening in south Africa: a cluster-randomized trial. Clin Infect Dis. 2022 Sep 14;75(5):849–56. doi: 10.1093/cid/ciab1047 34950944 PMC9477445

[36] Zhang C, Liu Y, Yao Y, Gong D, Lei R, Xia Y, et al. Tuberculosis infection among close contacts of patients with pulmonary tuberculosis in China: a population-based, multicentered study. Clin Microbiol Infect. 2024 Jun 6;30(9):1176–82. doi: 10.1016/j.cmi.2024.06.003 38851427

[37] Migliori GB, Nardell E, Yedilbayev A, D’Ambrosio L, Centis R, Tadolini M, et al. Reducing tuberculosis transmission: a consensus document from the World Health Organization Regional Office for Europe. Eur Respir J. 2019 Jun 5;53(6):1900391. doi: 10.1183/13993003.00391-2019 31023852

[38] Beeson E. The warning signs that should not be ignored. Penningthon. Manches cooper publisher.2017.

[39] Chiang SS, Brooks MB, Jenkins HE, Rubenstein D, Seddon JA, van de Water BJ, et al. Concordance of drug-resistance profiles between persons with drug-resistant tuberculosis and their household contacts: a systematic review and meta-analysis. Clin Infect Dis. 2021 Jul 15;73(2):250–63. doi: 10.1093/cid/ciaa613 32448887 PMC8427728

