# Supplementary material for: Active tuberculosis in household contacts of bacteriologically confirmed pulmonary tuberculosis patients: A multicenter study finding the ‘Missed One’ in Central Ethiopia
Source: PLoS One. 2025 Feb 18;20(2):e0316903. doi: 10.1371/journal.pone.0316903 (PMC11835242; doi:10.1371/journal.pone.0316903)
Supplement: S1 Fig — (DOCX) [file pone.0316903.s001.docx]

Bacteriologically confirmed PTB Index case join TB clinic

Declare HHCs and list them

TB focal screen index case & record full address and give to respective HEW

HEW appoint home visit

HEW screen HHCs for TB symptom(cough >2 weeks and other)

Symptomatic HHCs give sputum sample

TB negative/Asymptomatic adult HHCs advised to visit health facility when they fill any symptom

Children <15 years of age without TB symptom initiate TPT based on eligibility criteria

Laboratory investigation (Smear microscopy, Xpert ultra assay and TB culture

Confirmed TB case joins anti TB treatment

S 1 Fig .Tuberculosis screening algorithm for HHCs.
