# Supplementary material for: Active tuberculosis in household contacts of bacteriologically confirmed pulmonary tuberculosis patients: A multicenter study finding the ‘Missed One’ in Central Ethiopia
Source: PLoS One. 2025 Feb 18;20(2):e0316903. doi: 10.1371/journal.pone.0316903 (PMC11835242; doi:10.1371/journal.pone.0316903)
Supplement: Attachment — Submitted filename: Review comments.docx [file pone.0316903.s002.docx]

**Manuscript Number:** PONE-D-24-35318

**Title:** Active Tuberculosis in Household contacts of Bacteriologically confirmed Pulmonary Tuberculosis patients: A Multicentre Study Finding the ‘Missed One' in Central Ethiopia.

This manuscript provides very good information and clarifies further gap in the low economic countries like Ethiopia. The manuscript can be considered for publication after required minor corrections given below.

1. Abbreviate the short words like TB, HHC in its first appearance especially in Abstract.
2. In the abstract background section, please provide the clear intro and gaps in the area of research. The statement “The study was conducted to assess the yield of door-to-door TB household contact investigation among household contact of bacteriologically confirmed pulmonary TB cases in central Ethiopia” is better fit in to Methods section. A brief about the study gap is missing in the background section.
3. In Introduction section please correct the word contracted. “Worldwide in 2022, 10.6 million people contracted tuberculosis (TB)”. Should it be Contacted?
4. Please clarify on Household items you are targeting. What are the potential items in the Introduction session.
5. Please either provide reference for the procedure used to diagnose the TB or explain the procedure. Such as for Smear microscopy, Gene Xpert MTB/RIF Ultra, and/or culture test.
6. You have clearly given the inclusion criteria for the study subjects. Please also provide the exclusion criteria with proper justification.
7. Provide a clear definition for the index TB case. As the study is majorly focussed on index TB.
8. Statistical p values may require correction. Suggesting to use corrected p value. Such as Yates correction or Bonferroni correction. This will reduce the bias in the data.
9. Provide the message or benefit of study findings to the changes required in patient treatment, prescription, and recommendations to the clinicians in the discussion section and brief in conclusion.
10. None of the study area is 100% complete. Please provide the open research question, how much of the part this current study covered, and what is the gap in study. Give a clue to the researchers in similar field about the gaps and further research requirement.
