# Supplementary material for: Active tuberculosis in household contacts of bacteriologically confirmed pulmonary tuberculosis patients: A multicenter study finding the ‘Missed One’ in Central Ethiopia
Source: PLoS One. 2025 Feb 18;20(2):e0316903. doi: 10.1371/journal.pone.0316903 (PMC11835242; doi:10.1371/journal.pone.0316903)
Supplement: Attachment — Submitted filename: Response to reviewers.docx [file pone.0316903.s003.docx]

**Response to reviewers**

**Manuscript Number:** PONE-D-24-35318

**Title:** Active Tuberculosis in Household contacts of Bacteriologically confirmed Pulmonary Tuberculosis patients: A Multicentre Study Finding the ‘Missed One' in Central Ethiopia.

**Dear Editor and Reviewers**

We would like to thank you for your constructive and in-depth review and comments. We believe that the given comments and suggestions improved the quality of the paper.

**Specific Comments:**
**1.Introduction:**
References such as those on TB incidence and the 'missing millions,' could benefit from more recent data to reflect any changes in global trends.
**Response:** Thank you for the comment, we put a reference in the revised manuscript. Yes the data was updated based on the recent WHO global TB report .Page 1 line 72-73
**2.Methods:**
**1)**The study mentions using the Xpert Ultra assay and TB culture for diagnosis. It would be helpful to briefly describe the sensitivity and specificity of these methods.

**Response:** Thank you for the suggestion. In the revised manuscript**,** we described the sensitivity and specificity of Xpert Ultra assay and the lower detection limit for TB culture was included in method section page 6 line 199-202.

“Xpert Ultra assay has a sensitivity of 90%(95%CI:84-94) and specificity 96%(95%CI:93-98). Tuberculosis culture is highly sensitive with lower detection limit of 10-100CFU/ml”
**2)** Consider adding more details on how "presumptive TB" was defined during the household evaluations.

**Response:** Thank you for the important comment. We revised it, based on the given comment (line 181-184).
**3)** Actually its Spearman's rank correlation coefficient or Spearman's ρ, not "Spear’s man correlation".
**Response:** Thank you for the comment, we revised it accordingly.
**3. Results:**
1)The section detailing the correlation between the index case’s cycle threshold (Ct) values and household contacts is intriguing. It would be beneficial to include a brief explanation of why this correlation is important in the context of TB transmission.

**Response:** Thank you for the important suggestion. It is corrected accordingly. There is an increased risk of Mtb transmission to close contacts when the TB index case has a high bacillary load. Page 12 line 310-311
**2)**The comparison between DS-TB and MDR-TB households is valuable. However, the discussion of the similar yield in active TB detection between the two groups should address the potential for misclassification or under-diagnosis of MDR-TB in the HHCs.

**Response:** Thank you for the comment**.** We tried to discuss the finding in line 341-347.

Although the number of MDR-TB index patients in our study was fewer than the number of DS-TB index cases, we found no significant difference in active tuberculosis contact investigation yield among HHCs based on the drug resistance profile of the index case. A similar study finding was reported from Ethiopia (30). MDRTB patients are less likely to infect others than patients with drug-susceptible tuberculosis (31). This makes our finding welcome but it lacked statistical power to distinguish between the number of secondary cases in MDR-TB index cases HHCs versus DS-TB index case contacts.

**3)** Consider highlighting key findings in bullet points or using summary tables for improved clarity.

**Response:** Thank you for the suggestion. We tried to put key findings of the study at the end of result section. Page 12 line 309-315
**4)** Figure 1 is useful in showing the contact investigation cascade. However, it could be enhanced with a visual distinction between DS-TB and MDR-TB groups to make differences more apparent.
**Response:** We revised the figure as per the given comment and now the visualization is improved.
**4. Discussion:**
**1)**The limitations are acknowledged, such as the lack of clinical screening (e.g., chest X-rays). However, the authors could further discuss how the use of such diagnostic tools might alter the study's conclusions.

**Response:** The chest x–ray might increase the sensitivity of TB screening as it increase the number of presumptive TB case. It was addressed in page 13 line 379.
**2)**The study's impact on future TB control strategies could be emphasized more. For example, what specific recommendations can be made for integrating door-to-door TB investigations into existing public health frameworks?

**Response:** Incorporating door-to-door TB investigations into existing public health structures, such as utilizing health extension workers and women's community groups, could improve TB control strategies.

Please see conclusion part of revised manuscript page 14

This manuscript provides very good information and clarifies further gap in the low economic countries like Ethiopia. The manuscript can be considered for publication after required minor corrections given below.

1. Abbreviate the short words like TB, HHC in its first appearance especially in Abstract.

**Response:** Thank you for the suggestion and we made a revision accordingly

2. In the abstract background section, please provide the clear intro and gaps in the area of research. The statement “The study was conducted to assess the yield of door-to-door TB household contact investigation among household contact of bacteriologically confirmed pulmonary TB cases in central Ethiopia” is better fit in to Methods section. A brief about the study gap is missing in the background section.

**Response:** Thank you for the important comment. We included the current gap in the abstract background section of the revised manuscript.

3. In Introduction section please correct the word contracted. “Worldwide in 2022, 10.6 million people contracted tuberculosis (TB)”. Should it be Contacted?

**Response:** Yes it was corrected with appropriate word

4. Please clarify on Household items you are targeting. What are the potential items in the Introduction session?

**Response:** We have targeted household items like number of living room, number of windows in the household.

5. Please either provide reference for the procedure used to diagnose the TB or explain the procedure. Such as for Smear microscopy, Gene Xpert MTB/RIF Ultra, and/or culture test.

**Response:** Yes the references were there in the method section page (reference number 19 and 20)

6. You have clearly given the inclusion criteria for the study subjects. Please also provide the exclusion criteria with proper justification.

**Response:** Thank you for the comment and it was described in method section page 5 line 161-162.

7. Provide a clear definition for the index TB case. As the study is majorly focussed on index TB.

**Response:** Thank you for the comment and it was included in method section page 5 line 154-156.

8. Statistical p values may require correction. Suggesting to use corrected p value. Such as Yates correction or Bonferroni correction. This will reduce the bias in the data.

**Response:** Thank you for the important comment we addressed the comments as follow Groups were compared using a Chi-square test with Yates correction of continuity. A two-tailed p value of less than 0.05 was considered as statistically significant. See Result section page 11 Table 3 and method section

9. Provide the message or benefit of study findings to the changes required in patient treatment, prescription, and recommendations to the clinicians in the discussion section and brief in conclusion.

**Response:** To ensure early detection and treatment of TB, healthcare providers must screen HHCs of patients with bacteriologically confirmed TB in timely manner. see page 14 line 392-393

10. None of the study area is 100% complete. Please provide the open research question, how much of the part this current study covered, and what is the gap in study. Give a clue to the researchers in similar field about the gaps and further research requirement.

**Response:** We suggest conducting additional research using whole genome sequencing to identify the actual index case and understand the transmission dynamics.

Please see page 14 line 382-383.
