# Supplementary material for: Active tuberculosis in household contacts of bacteriologically confirmed pulmonary tuberculosis patients: A multicenter study finding the ‘Missed One’ in Central Ethiopia
Source: PLoS One. 2025 Feb 18;20(2):e0316903. doi: 10.1371/journal.pone.0316903 (PMC11835242; doi:10.1371/journal.pone.0316903)
Supplement: Attachment — Submitted filename: Response_to_Reviewers_auresp_2.docx [file pone.0316903.s004.docx]

**Response to Reviewers**

**Reviewer #1:** All my recommendations in minor revision have been addressed. The manuscript can be proceeded for next steps in publication.

**Response:** Dear reviewer thank you for your in-depth comments and review and we are lucky to address your comments.

**Reviewer #2:** 1. The definition of diagnostic is undoubtable following the guideline in line 141-145. "An active pulmonary TB case is a TB case confirmed by either smear microscopy, Gene Xpert MTB/RIF Ultra, or a TB culture test. Bacteriologically confirmed pulmonary tuberculosis: case referred to a pulmonary TB patient with biological specimen positive by Acid-Fast Bacilli (AFB) smear microscopy, Xpert MTB/RIF assay or TB culture, indifference with drug susceptibility profile."

Now what worrying is the diagnosis of bacteriologically confirmed pulmonary tuberculosis in HHCs, which means that the test may not covered all of three methods of AFB, Xpert, and culture simultaneously, it is a "OR" in your diagnostic criteria. The results may include false positive of dead bacteria patients, on the basis of the screening produce also not included the chest X-ray, which may bias the results.

This should be discussed and should be put forward in limitation, not only simply narrate in limitation line 389-390 "We did not use clinical screening like chest X-rays which might increase the sensitivity of screening. "

**Response:** Thank you for your comment. In this study from presumptive HHc sample we did (AFB) smear microscopy, Xpert ultra assay and TB culture test simultaneously. Page 6 line 184-185.It was corrected as follows in the revised manuscript. All the 13 active tuberculosis cases found from HHCs were culture positive. line 245-246

2.Line 292-294 "Whereas among thirty presumptive household contacts of MDR-TB patients, 2 (6.67%; 95% CI: 1.16- 23.51) were MTB detected Rifampicin resistance was not detected." this need to discuss in depth, is any other evidence or report that the MDR-TB HHCs not infected with MDT-TB strains, it should be addressed and clarify the primary and secondary drug resistance in this case.

**Response:** Thank you for the good comment. Close contacts of MDR-TB patients are more likely to contract drug-resistant TB (DR-TB). Meanwhile, contradicting evidence has been reported from many studies regarding the risk of TB in close contact of drug-susceptible and MDR-TB patients (reference numbers 8,9 and 10). line 91-92.

In a single household, two individuals can have been exposed to different M. tuberculosis strains from the surrounding community. Studies have described that a significant number of contacts living in the same household possess different drug-resistance profiles in contrast to the index patients (10, 39). line 380-385

For the culture-positive isolates, due to resource shortage, we are considering performing phenotypic DST in the future.

**3.** line 300 "There was a positive correlation between the ct values of the index case and the corresponding HHC ct) value at each probe ", This bracket seems superfluous.

**Response:** Thank you for the comment. We have edited the unnecessary bracket in the revised version.line 300

**4**. last time comment "Consider highlighting key findings in bullet points or using summary tables for improved clarity.

Author Response: Thank you for the suggestion. We tried to put key findings of the study at the end of result section. Page 12 line 309-315"

Lets make it clear that authors could embeded the key findings in the article, not only list and summary in the back of results, which is a bit redundant and may overlap with the discussion. Author should also be associated with the journal's editorial policy and format.

**Response:** Thank you for the nice comment. Of course the key findings were addressed in the first paragraph of the discussion section. line 318-327

We had read the plos one journal editorial policy and format but we were not able to get the specific section where the key finding(Author summary) of the study should have to put like other plos journals (e.g plos complex system,plos neglected tropical disease submission guideline which recommend author summary next to the abstract section ).Based on the journal editorial policy we are not able to add author summary.
